# Repeated AAV9 Titer Determination in a Presymptomatic SMA Patient with Three *SMN2* Gene Copies – A Case Report

**DOI:** 10.3233/JND-221659

**Published:** 2024-03-05

**Authors:** Astrid Eisenkölbl, Manuel Pühringer

**Affiliations:** Kepler University Hospital, Linz, Austria

**Keywords:** Spinal muscular atrophy, gene transfer therapy, AAV9 antibody titer, onasemnogene abeparvovec, floppy infant

## Abstract

Adeno-associated viruses (AAV) are well-suited to serve as gene transfer vectors. Onasemnogene abeparvovec uses AAV9 as virus vector. Previous exposure to wild-type AAVs or placental transfer of maternal AAV antibodies, however, can trigger an immune response to the vector virus which may limit the therapeutic effectiveness of gene transfer and impact safety. We present the case of a female patient with spinal muscular atrophy (SMA) and three survival motor neuron 2 (*SMN2*) gene copies. The infant had elevated titers of AAV9 antibodies at diagnosis at 9 days of age. Being presymptomatic at diagnosis, it was decided to retest the patient’s AAV9 antibody titer at two-weekly intervals. Six weeks after initial diagnosis, a titer of 1:12.5 allowed treatment with onasemnogene abeparvovec. The presented case demonstrates that, provided the number of *SMN2* gene copies and the absence of symptoms allow, onasemnogene abeparvovec therapy is feasible in patients with initially exclusionary AAV9 antibody titers of >1:50.

## INTRODUCTION

Spinal muscular atrophy (SMA) is a rare autosomal recessive neurodegenerative disease, affecting 1 in 10000 live births [1]. SMA is characterized by a dysfunction or loss of the survival motor neuron 1 (*SMN1*) gene resulting in a critical deficiency of SMN protein. A lack of SMN protein results in impaired alpha motor neuron development leading to progressive muscle weakness, atrophy and paralysis [2]. The paralogous *SMN2* gene also produces functional SMN protein, albeit at much lower levels. SMA disease severity thus depends on the number of *SMN2* copies [3].

Gene replacement therapy for SMA uses adeno-associated virus 9 (AAV9) as a gene transfer vector. The vector delivers the genetic material into the episomes of motor neuron cell nuclei without integration into the chromosomes [4]. Because the virus capsid –the protein shell that encloses the viral genome –of the wild-type AAV is highly homologous to the vector capsid, any pre-existing immunity to the wild-type virus can trigger an immune response to the vector virus thus potentially limiting the therapeutic effectiveness of gene transfer and impacting on safety [5, 6]. It is therefore essential that candidates are tested for AAV antibody titers.

If the newborn with SMA turns out to have a high AAV9 titer (above 1:50), important questions arise: Can therapy be delayed until antibody titers fall or should the waiting time be bridged with alternative therapies? Can antibody titers be temporarily suppressed to allow for gene therapy? Is breastfeeding allowed when the mother carries AAV9 antibodies? We report the case of a presymptomatic newborn girl with SMA and three *SMN2* copies and review the literature for evidence to answer these essential questions.

## CASE DESCRIPTION

The girl was diagnosed with SMA already on her 5^th^ day of life due to a positive family history. Her older brother was diagnosed with SMA type 2 just half a year earlier and has since then been treated with nusinersen. Both children have three *SMN2* copies. Table 1 shows an overview of demographic and clinical details of the girl. The girl was breastfed from birth and over 13 months. As she was planned to be treated with onasemnogene abeparvovec (Zolgensma^®^, Novartis Gene Therapies), the anti-AAV9 antibody titer was measured (day −40 from onasemnogene abeparvovec administration). This test was performed using the Viroclinics Anti-AAV9 human immunoglobulin (Ig) G enzyme-linked immunosorbent assay (ELISA) method VC-M178-2, which is the same test as the one used in the STR1VE-EU study [7] and described in detail by Tijsma ASL, et al. [8]. This laboratory performs central routine AAV9 antibody titer testing to determine patients’ suitability for onasemnogene abeparvovec therapy in clinical practice globally except US and Japan.

**Table 1:** Clinical and demographic overview

Sex	Female
Number of *SMN2* copies	3
Family history of SMA	Yes
Presence of symptoms	Presymptomatic
AAV9 antibody titer	1^st^ titer on 4 March 2021: 1:200
	2^nd^ titer on 18 March 2021: 1:100
	3^rd^ titer on 1 April 2021: 1:12.5
Age at onasemnogene abeparvovec administration	7 weeks
Weight at onasemnogene abeparvovec administration	4.88 kg
Side effects	Thrombocytopenia, feeding intolerance, mild hepatic dysfunction
Symptoms/signs related to hepatic dysfunction	Asymptomatic
Family history of liver disease	None
Duration of steroid	2 months
Method of respiratory support	None
Presence of scoliosis	No
Motor achievements at last evaluation	Walks independently, climbs stairs
Age at last evaluation	16 months
Breastfeeding	Yes

AAV9, adeno-associated virus 9.

**Fig. 1:**
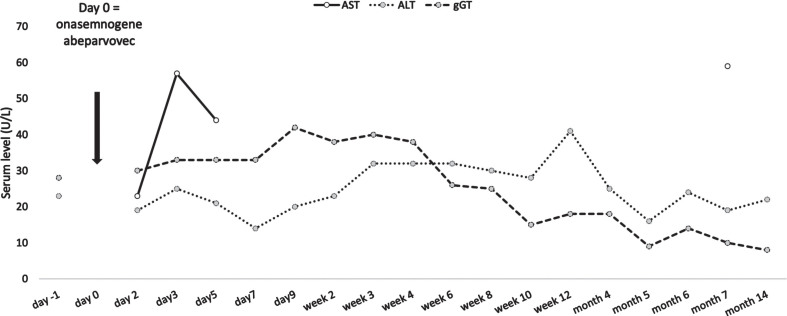
Liver function over time. ALT, alanin-aminotransferase; AST, aspartate-aminotransferase; gGT, gamma-glutamyl transferase.

**Fig. 2:**
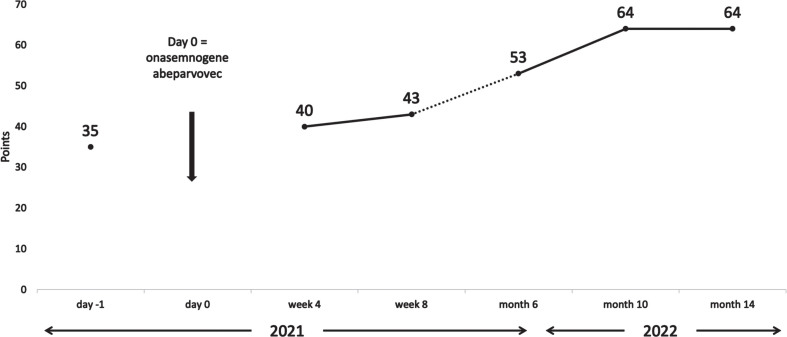
Development of CHOP-INTEND over time. CHOP-INTEND, Children’s Hospital Of Philadelphia Infant Test Of Neuromuscular Disorders.

The initial test result showed a titer of 1:200. We allowed for a retest two weeks later (day −26 from onasemnogene abeparvovec administration) which showed a decreased antibody titer of 1:100. A final screening test after two more weeks was negative with a titer of 1:12.5 (day −12 from onasemnogene abeparvovec administration). The mother’s AAV9 antibody titer was not measured. After detailed pretreatment consultation and all the required examinations, prednisone at a dose of 1 mg/kg was started (day −1 from onasemnogene abeparvovec administration). At the age of 7 weeks, the girl received gene transfer therapy (day 0). Onasemnogene abeparvovec was administered at the label-recommended dose of nominal 1.1×10^14^ vector genomes/kg, which she tolerated well. She could be discharged 9 days later. A slight thrombocytosis (minimum level 223 g/L on day 5) and mild iatrogenic anemia (minimum 9.1 mg/dL on day 7) were observed with both levels back to within their normal range as of week 2. Transaminases were not markedly elevated (Fig. 1) and the prednisone dose could be tapered by week 4 and stopped in week 7. For a few weeks the girl did not thrive optimally, due to insufficient drinking, which resolved after stopping prednisone. In the months following treatment she had regular follow-up visits and showed an age-appropriate development. At the age of 12 months, having attained independent sitting, crawling, and upright standing, she started walking independently, reaching the maximum CHOP-INTEND score of 64 approximately 10 months after gene transfer treatment. Figure 2 shows the development of the CHOP-INTEND score over time.

## CASE DISCUSSION AND REVIEW OF THE LITERATURE

The presented presymptomatic infant had three *SMN2* copies. The AAV9 antibody titer was in the borderline range [4] and was expected to fall rapidly to below the 1:50 threshold [9]. Under tight-meshed monitoring, it was feasible to delay treatment until eligibility for onasemnogene abeparvovec was confirmed.

Immune reactions to gene therapies can happen in three distinct ways. Firstly, as in our case, antibodies to AAV9 or related antigens can be present in the newborn through transfer from the mother. Secondly, immunity to AAVs could have formed in response to previous exposure of the child to an AAV or a related antigen, which is more likely in older children. Thirdly, the immune system can react to the vector, the vector components, or the transgene of the drug product whether or not it was previously exposed to an AAV or related antigen. In our review of the basic immune processes, we will focus on the situation of maternal transfer of AAV antibodies and refer to literature [4, 6, 10, 11] for further reading on the immunogenicity of AAV vectors for gene transfer in general.

Mothers can transfer antibodies transplacentally (IgG isotype) in a finite amount or via their breast milk (IgA isotype) as a steady stream [12]. IgA antibodies remain in the gastrointestinal tract and have no effect on the immune response to parenterally administered gene therapies. IgG antibodies are present in the bloodstream and can therefore protect the newborn against infections, but can also elicit immune responses to the vector, the vector components, and the transgene products [10, 12]. Maternally transferred IgG antibody titers typically decline over a period of 4 to 12 months through metabolization, whereby higher titers persist for a longer time [11, 13–18]. Importantly, maternal exposure to wild type virus infection, as is the case of maternal AAV9 antibodies, typically results in higher antibody transfer rates into children than maternal exposure to vaccines [19]. Interestingly, girls have been reported to have lower maternal antibody titers [20].

Given the marked likelihood of seropositivity in newborns through maternal antibody transfer and the important implications on efficacy and safety of gene transfer therapy, it is essential that candidates are tested for AAV antibody titers. Assays currently used to detect neutralizing and non-neutralizing anti-AAV antibodies include ELISA-based binding assays [4]. Antibody titers are determined by serial dilution of sample until no further fluorescent signal can be detected. Depending on the specific assay used, cut-off levels above which a sample is considered antibody positive are determined. For AAV9, cut-off levels of ≥1:25 dilution or >1:50 dilution have been used in clinical trials of onasemnogene abeparvovec [4, 11] and a titer cut-off of 1:50 is currently recommended in the onasemnogene abeparvovec label [9].

The prevalence of anti-AAV9 seropositivity differs by geographical location. However, there is a known cross-reactivity of AAV9 antibodies with other, more frequent AAV serotypes. A seroprevalence study of serum IgG determined by ELISA and neutralizing factors against AAV types 1, 2, 5, 6, 8, and 9 in the healthy population showed anti-AAV1 total IgG in 67% of probands, anti-AAV2 in 72%. The prevalence of IgG for anti-AAV5 (40%), anti-AAV6 (46%), anti-AAV8 (38%), and anti-AAV9 (47%) were also high [21].

### Can gene therapy be delayed or should the waiting time be bridged with alternative therapies?

To date, the evidence around waiting and retesting of patients with elevated AAV9 antibody titers is still based on a very limited number of patients and further research is needed to develop standardized procedures. Systematic screening of infants with SMA type 1 and their biologic mothers within the framework of onasemnogene abeparvovec clinical trials and managed access programs showed that 7.7% of children and 14.8% of mothers had exclusionary antibody titers of >1:50 on their initial screening tests. On the final screening tests, titers of 5.6% of children remained elevated [11]. These numbers are from type 1 SMA patients, who are expected to be diagnosed within the first 6 months after birth [22]. In general, maternal antibodies have been observed to reach undetectable levels at 4 to 6 months after birth [11, 17, 18]. In infants identified as at risk for SMA in newborn screening, the prevalence of high anti-AAV antibody should be expected to reflect that in the adult female population of the respective geographic region [21].

It was observed, however, that decreases in AAV9 antibody titers may be limited to very young infants up to 4 months of age. In older children, decreases in anti-AAV9 antibody titers were not detected in the above investigation [11]. This indicates that a non-negligible percentage of infants with initially elevated AAV9 antibody titers should be able to receive onasemnogene abeparvovec after a certain lag period. If retesting is considered, the children need to be monitored closely so that any onset of symptoms is immediately detected and appropriate action can be taken.

Clinically, treating physicians need to decide on a case-by-case basis when and how to bridge therapy until eligibility for gene therapy has been confirmed. In general, there is strong evidence that infants with three *SMN2* copies require immediate treatment [23] and the Cure SMA working group also recommends immediate treatment for infants with four *SMN2* copies [24]. This recommendation implies a need for bridging therapy in infants with elevated AAV9 antibody titers planned for treatment with onasemnogene abeparvovec. In clinical practice, several questions remain unanswered. Firstly, as established earlier, in infants identified with SMA during newborn screening, elevated AAV9 antibody titers are expected to be caused by maternal transfer and are therefore expected to fall below the level of 1:50 within the first 6 months of life. However, as explained earlier, the time it will take for the AAV9 titer to fall, will be longer in infants with a very high initial titer. It is currently unknown, at which initial AAV9 antibody titer a bridging therapy should be advised. It is also unknown, what could be considered an ‘acceptable waiting time’ before initiation of a bridging therapy, since there are no routinely established biomarkers. Neurofilament in the cerebrospinal fluid, serum creatinine, and muscle-specific miRNAs have been suggested as reliable biomarkers [25]. Of these, only serum creatinine is currently routinely assessed, which is insufficient as a stand-alone biomarker.

Secondly, treatment with any approved and reimbursed therapy will take time. Nusinersen, for instance, requires four loading doses over 63 days [26], which would have to be followed by a wash-out phase; only then could gene therapy be administered. It is unknown, if this procedure should also be followed in infants with a low initial AAV9 antibody titer, which can be expected to fall below the 1:50 threshold long before the full loading procedure can be completed. The consequences of earlier discontinuation of the bridging treatment are also not sufficiently understood. Therefore, further research into the practical aspects of bridging urgently needs to be conducted. This is especially important, since similar questions arise for situations where there is a lag between SMA diagnosis and the payers’ decision to reimburse onasemnogene abeparvovec.

### Can antibody titers be temporarily suppressed to allow for gene therapy?

While seropositivity resulting from filial infection may be transiently suppressed using rituximab, sirolimus or eculizumab, clinical evidence for such an approach is still immature [4].

### Is breastfeeding allowed when the mother carries AAV9 antibodies?

Seropositivity in infants can occur through maternal antibody transfer through the placenta [4, 21, 27]. Transplacental IgG has an approximate half-life of 6 weeks and the resulting passive immunity to AAV9 in infants would thus be expected to be rapidly lost [17, 27]. Transfer of IgA via breast milk is considered negligible [28] and breastfeeding should not have a negative impact on the possibility of onasemnogene abeparvovec therapy [11], as was also seen in the presented case.

## CONCLUSIONS

The presented patient case demonstrates that provided the number of *SMN2* copies allows and monitoring of the patient ensures continued absence of symptoms, onasemnogene abeparvovec treatment may be feasible even in patients with initially exclusionary AAV9 antibody titers of >1:50. Systematic studies are needed to develop standardized decision guidelines for such scenarios.

## ETHICAL STATEMENT

The parents provided written informed consent for the publication of the present patient case.

## CONFLICT OF INTEREST

A.E. has received speaker’s honoraria and advisory fees from Novartis, Biogen, Roche, and Sarepta. M.P. has no conflicts of interest to declare.

## FUNDING

None.
